# Association between sleep duration and child development: the Japan Environment and Children’s Study

**DOI:** 10.3389/fpubh.2026.1736659

**Published:** 2026-07-08

**Authors:** Toshio Masumoto, Hiroki Amano, Shinji Otani, Youichi Kurozawa, Akemi Morita

**Affiliations:** 1 Division of Health Administration and Promotion, Department of Social Medicine, Faculty of Medicine, Tottori University, Tottori, Japan; 2 International Platform for Dryland Research and Education, Tottori University, Tottori, Japan

**Keywords:** infant, JECS, mental development, sleep duration, sleep environment

## Abstract

Few studies have examined the association between total sleep duration and general child development, as well as the environmental factors influencing sleep duration among children. To elucidate this issue, we investigated the association between sleep duration, child development, and the sleep environment. We used data from an ongoing nationwide prospective birth cohort study in Japan. Developmental delays were measured using the Ages and Stages Questionnaires, third edition (ASQ-3). Sleep duration was assessed using a questionnaire completed by the children’s caregivers. To assess the associations, we performed logistic regression and generalized linear mixed model analysis. Shorter sleep duration (< 10 h) at multiple time points was associated with an increased likelihood of developmental delay at 3 years of age (Sleep@6 m: Odds ratio (OR) 1.11, *p* < 0.001; Sleep@1y: OR 1.09, *p* = 0.01; Sleep@3y: OR 1.15, *p* < 0.001). These associations were still observed after adjusting for individual characteristics and relevant covariates (coefficient: 0.054; *p* < 0.001). In analyses examining the sleep environment, sleep location was associated with shorter sleep duration, whereas other environmental factors showed no significant associations (coefficient: 0.13; *p* = 0.049). These findings suggest that shorter sleep duration during early childhood is associated with developmental outcomes and that sleep location may be related to sleep duration. Further studies are needed to clarify the underlying mechanisms and potential bidirectional relationships involved.

## 1 Introduction

Sleep is an essential behavior, and sufficient and good-quality sleep is important for human health. Previous studies in adults have shown that insufficient sleep is associated with physical illnesses, such as hypertension and diabetes, and mental illnesses, such as depression and self-harm (1–6). In addition, many studies have reported the negative health impacts of insufficient sleep in children. For example, insufficient sleep has been reported to be associated with obesity, mental health disorders such as anxiety and depression, and poor emotion regulation in children (7–9). Therefore, sufficient and good-quality sleep is important for maintaining health in both children and adults.

When assessing the health impacts of sleep, it is important to consider both sleep quality and total sleep duration. Regarding total sleep duration, the National Sleep Foundation has established age-specific recommendations (10), with the recommended sleep duration decreasing in an age-dependent manner. A previous study reported an increase in the proportion of children with insufficient sleep duration (11). Between 10 and 35% of parents report sleep problems in their infants, including insufficient sleep duration and nighttime awakening (12–15). As neuronal plasticity and the construction of neuronal circuits are particularly active during early life, insufficient sleep during infancy could potentially have a long-term effect on child development (16). In addition to total sleep duration, sleep quality encompasses multiple dimensions, such as nighttime awakening, sleep onset, and the sleep environment. Nighttime awakening in early life has been reported to be related to child development (17, 18). For example, a Norwegian birth cohort study reported that shorter total sleep duration, prolonged sleep onset, and frequent nighttime awakenings among 24-month-old children were associated with a greater risk of concurrent social–emotional problems in toddlerhood (7). In addition, children who sleep less than 11 h or awaken three or more times per night at 18 months of age have been reported to exhibit greater emotional regulation difficulties at 5 years of age (8). Consistent results have been reported regarding the association between nighttime awakenings and child development. However, the association between total sleep duration in early life and subsequent developmental outcomes remains controversial. A recent systematic review reported that the evidence remains inconclusive regarding the relationship between total sleep duration and child development because of the limited sample sizes (less than 1,000) of previous studies (18). Moreover, few studies have examined this association using repeated measurements while accounting for individual differences, such as developmental trajectories and sleep patterns. Consequently, the association between total sleep duration and child development remains unclear.

Total sleep duration and sleep quality are associated with the external sleep environment, such as physical conditions and parent–child relationships. To prevent insufficient sleep duration and poor sleep quality, it is important to identify the factors affecting sleep duration and sleep quality. Previous studies have reported that risk factors for sleep disruption include infantile colic and environmental noise (53). In addition, parent–child bed-sharing and room-sharing have been reported to influence sleep duration and sleep quality (12, 19–21). However, the association between the sleep environment and sleep duration has not been sufficiently examined. As insufficient sleep has been associated with adverse child health outcomes, it is important to investigate factors related to insufficient sleep and maintain an environment that enables children to achieve sufficient sleep duration.

As mentioned above, few studies have examined the association between total sleep duration in early life and general child development using repeated measurement data that adjust for individual differences, and the relationship between the sleep environment and sleep duration in early life is not well understood. To address this issue, we investigated the association between total sleep duration and developmental outcomes while adjusting for individual differences using large-scale repeated measurement data. In addition, we assessed the association between the sleep environment and shorter sleep duration to elucidate the factors affecting sleep duration in children.

## 2 Methods

### 2.1 Participants and data source

The aim of the Japan Environment and Children’s Study (JECS), which is an ongoing prospective birth cohort study that began in 2011, is to evaluate the effects of various environmental factors on the health and development of children (22, 23). The JECS protocol was reviewed and approved by the Ministry of the Environment’s Institutional Review Board on Epidemiological Studies and the ethics committees of all participating institutions. In the current study, the JECS dataset (jecs-ta-20190930) that was released in October 2019 was used for analysis. The dataset does not contain any identifiable patient information. Written informed consent was obtained from all study participants.

**Figure 1:**
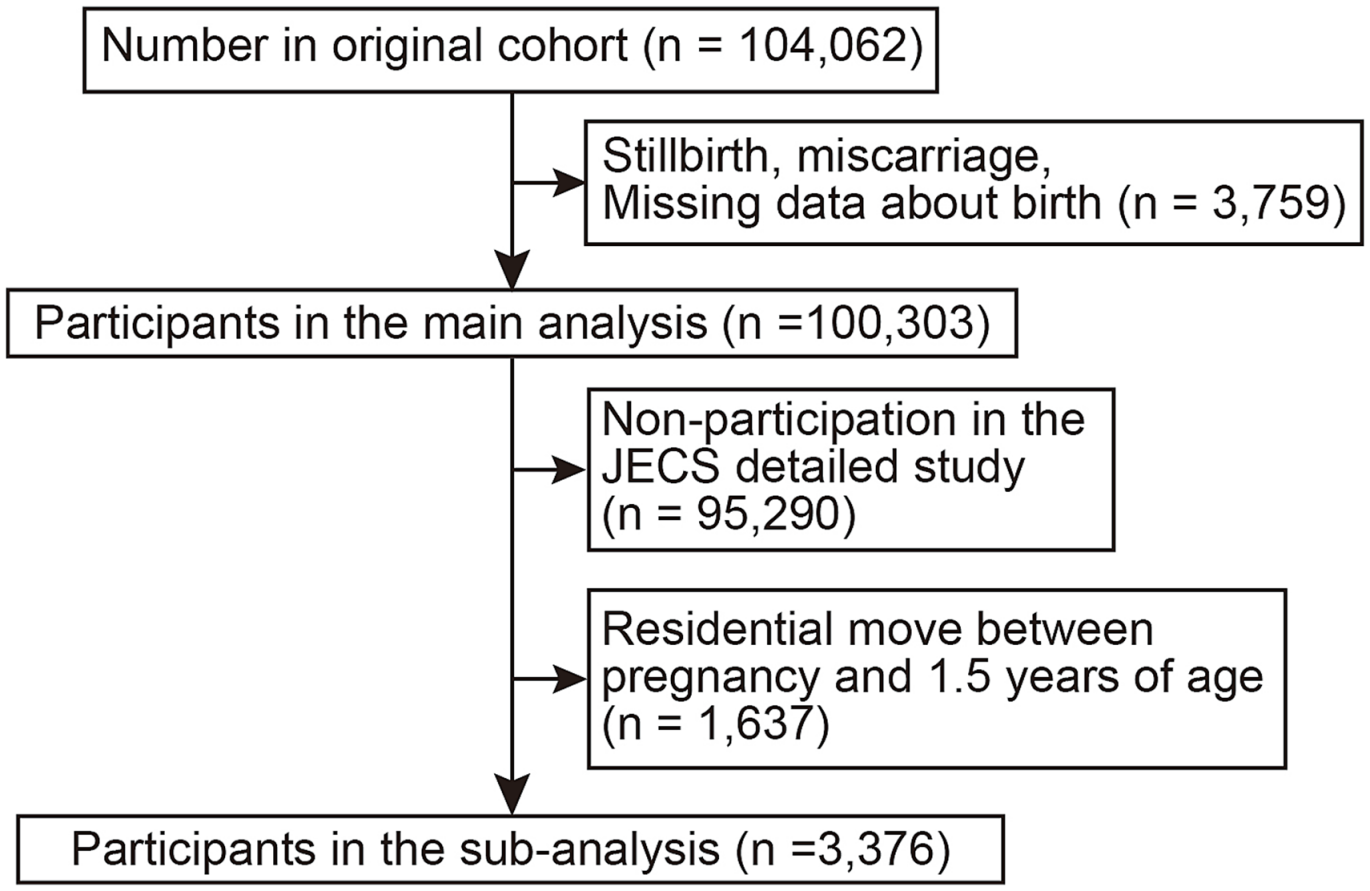
Participant flow diagram for the current study.

We administered a questionnaire to each participant of the study during the first trimester of pregnancy, either in person or by post, after explaining the purpose and procedures of the study at recruitment. Follow-up questionnaires were administered in person or by post during the second and third trimesters and at 1 month postpartum, coinciding with scheduled medical checkups. Subsequent follow-up questionnaires were sent by post at 6, 12, 18, 24, 30, and 36 months postpartum. In total, 104,062 fetal records were registered across 19 prefectures in Japan (Aichi, Chiba, Fukushima, Fukuoka, Hokkaido, Hyogo, Kanagawa, Kochi, Kumamoto, Kyoto, Miyagi, Miyazaki, Nagano, Okinawa, Osaka, Shiga, Toyama, Tottori, and Yamanashi) between January 2011 and March 2014. We excluded 3,759 participants due to stillbirth, miscarriage, or missing birth data. Consequently, 100,303 participants were included in the main analysis examining the association between sleep duration and child development. In the sub-analysis examining the association between the sleep environment and sleep duration, we excluded 95,290 participants who were not included in the Detailed Study (24). The Detailed Study was a randomly selected sub-cohort of approximately 5,000 children from the overall JECS cohort and included more detailed assessments of environmental factors and questionnaire-based information. Therefore, participants who were not included in the Detailed Study were excluded from the sub-analysis (Figure 1).

We also excluded children who had moved residence between pregnancy and 1.5 years of age. A residential move was defined as any reported change in the child’s primary place of residence during this period. This exclusion was applied because the sub-analysis aimed to evaluate the association between the residential sleep environment, as assessed in the Detailed Study, and sleep duration; changes in residence during early life could lead to misclassification of the relevant sleep environment. If a child had more than one residence, such as in cases of parental separation or divorce, classification was based on the residence reported as the child’s primary place of residence in the questionnaire. Cases with an unclear residential history were excluded if a residential move was reported. We further excluded 1,637 participants who had changed residence between pregnancy and 1.5 years of age, leaving 3,376 participants for the sub-analysis (Figure 1).

### 2.2 Sample size and statistical power

As this study was a secondary analysis of an existing nationwide birth cohort, the sample size was determined by the number of eligible participants with available exposure, outcome, and covariate data rather than by an *a priori* sample size calculation. We included all eligible participants to maximize statistical precision. The adequacy of the sample size was assessed based on the number of participants included in the main analyses, the number of outcome events, and the precision of the estimated associations, as reflected by the 95% confidence intervals.

### 2.3 Ethical approval

The JECS protocol was reviewed and approved by the Ministry of the Environment’s Institutional Review Board on Epidemiological Studies and the ethics committees of all participating institutions (No. 100910001). The JECS was conducted in accordance with the Declaration of Helsinki and other relevant national regulations and guidelines. Written informed consent was obtained from all participants involved in the study.

### 2.4 Measurement of child development

Data from the Ages and Stages Questionnaires, third edition (ASQ-3), completed at 0.5, 1, 1.5, and 3 years after birth as part of the JECS questionnaires, were used to assess child development (25). Cutoff values for the Japanese version of the ASQ-3 were used (26). ASQ-3 scores were divided into five categories: Communication, gross motor function, fine motor function, problem-solving, and personal–social. General developmental delay was defined as having scores below the cutoff values in more than one category. For analysis examining the association between shorter sleep duration and developmental delay in each category, developmental delay was defined as a score below the cutoff value for the respective category. For the generalized linear mixed model analysis, we used the number of developmental delay categories in which the score was below the cutoff value.

### 2.5 Measurement of sleep duration and sleep environments

For the measurement of sleep duration at 0.5, 1, 1.5, and 3 years of age, we used the following question to collect responses from caregivers: “Please report the time your child slept yesterday.” When the child slept, caregivers checked their sleep status every 30 min. We defined total sleep duration as the summation of responses to the sleeping time questionnaires. Nighttime awakening was assessed using questionnaire responses. A nighttime awakening was defined as a caregiver report that the child awoke between 9:00 p.m. and 6:00 a.m. Sleep duration was categorized based on age-specific recommended sleep duration guidelines (10). The recommended sleep durations were as follows: 12–15 h at 0.5 years of age, 11–14 h at 1 and 1.5 years of age, and 10–13 h at 3 years of age. At each assessment age, children whose sleep duration was below the lower limit of the age-specific recommended range were classified as having shorter sleep duration, while those whose sleep duration was within or above the recommended range were classified as having recommended or longer sleep duration.

Sleep environment variables included animal entering the bedroom (yes/no), environmental noise (yes/no), sleep disturbances due to noise (yes/no), the child’s sleeping place (with parents in the same bed or with parents in another bed or in a different room), the child’s sleep posture (supine/prone), scheduled bedtime (yes/no), and asthma (yes/no).

### 2.6 Confounding factors for the questionnaire data

A number of potential confounding factors related to child development were included, namely maternal age, maternal body mass index, marriage status, number of deliveries, gestational age, smoking status, medical history [attention deficit hyperactivity disorder (ADHD)], learning disorder, autism spectrum disorder, diabetes, and other developmental disorders, autism spectrum quotient (AQ10) (27, 28), gestational diabetes, household income, and educational level. A number of potential confounding factors for child outcomes were examined, including parity, sex, birth weight, and nursery attendance. These confounding factors were selected after assessing the results of previous studies on child development (29, 30).

### 2.7 Statistical analysis

Adjusted odds ratios (ORs) were calculated using binomial logistic regression analysis. Briefly, a model was constructed using binomial logistic regression analysis with delayed development as the dependent variable and confounding factors as independent variables. The model was then constructed with delayed development as the dependent variable and confounding factors significantly associated with delayed development as independent variables.

The relationship between shorter sleep duration and developmental delay was assessed using a generalized linear mixed model with a Poisson link function to account for individual differences arising from repeated measurements. The model included a random effect to account for differences between participants. The number of developmental delay categories was the dependent variable, and two sleep duration categories (e.g., 1 = shorter sleep duration; 0 = recommended or longer sleep duration) were the independent variables. Sleep duration and covariates were included as fixed effects. A random intercept for each child was included to account for within-child correlations arising from repeated outcome measurements. Coefficients, their standard errors, and *p*-values for the generalized linear mixed model were calculated.

The relationship between shorter sleep duration and the sleep environment was assessed using a generalized linear mixed model with a Poisson link function to account for individual differences arising from repeated measurements. “Shorter sleep duration” (yes = 1/no = 0) was the dependent variable, and the explanatory factors were animal entering the bedroom (yes = 1 / no = 0), noisy environment (yes = 1 / no = 0), sleep disturbances caused by noise (yes = 1 / no = 0), sleeping place (without parents = 2 / with parents in another bed = 1 / with parents in the same bed = 0), “sleep posture” (other = 1 / supine = 0), scheduled bedtime (yes = 1 / no = 0), and asthma (yes = 1 / no = 0). Coefficients, their standard errors, and *p*-values for the generalized linear mixed model were calculated. When sleeping place was associated with shorter sleep duration, *post hoc* pairwise comparisons among sleeping place categories were conducted using estimated marginal means with Tukey’s adjustment.

As a sensitivity analysis to address the influence of missing values, we used a multiple imputation method. Briefly, 100 simulated datasets were generated using the mice package in R (31). Each simulated dataset was analyzed using logistic regression analysis, and the estimates were combined using pooling rules (32).

To assess multicollinearity, we calculated the variance inflation factor and checked for values < 10. The variance inflation factor values were as follows: maternal age, 1.03; marriage status, 1.06; medical history (ADHD), 1.01; medical history (learning disorder), 1.01; medical history (other conditions), 1.00; smoking status, 1.08; delivery method, 1.10; AQ10: 1.00; multiparity, 1.13; gestational age, 1.36; birth weight, 1.40; sex, 1.01; education, 1.51; household income, 1.37; nighttime awakening, 1.51; and sleep duration, 1.13. In addition, we report a correlation matrix of key covariates and full regression coefficients for Models 1 and 2 in the Supplementary Information to aid interpretation (Supplementary Table 2).

Statistical analyses and graphing were performed using R software version 4.1.1 with the AER, tidyverse, nnet, ggplot2, mice, lme4, glmmTMB, and glmmML packages (33–36). Significance was defined as a *p*-value of < 0.05, and 95% confidence intervals were calculated.

## 3 Results

The general characteristics of participating mothers and children and the sleep characteristics of children are summarized in Tables 1, 2. Regarding sleep characteristics, we defined shorter sleep duration, recommended sleep duration, and longer sleep duration based on age-specific sleep duration recommendations proposed by the National Sleep Foundation (10). The proportion of children who did not meet the recommended sleep duration was highest at 0.5 years of age compared to all other ages. The proportion of children with the recommended sleep duration increased from 0.5 to 1.5 years of age and remained relatively stable at 3 years of age (Table 2).

**Table 1:** Participant characteristics.

Characteristics	Total	Number	Percentage of total 100%
Maternal characteristics			
Age	x < 20	1,069	1.07
	20 ≤ x < 30	37,238	37.13
	30 ≤ x < 40	52,375	52.22
	40 ≤ x	3,359	3.35
	NA	6,262	6.24
Number of previous deliveries	x = 0	39,564	39.44
	x ≥ 1	58,320	58.14
	NA	2,419	2.41
Gestational age	Full-term birth	94,507	94.22
	Preterm or post-term delivery	5,796	5.78
Smoking at MT1	No	57,233	57.06
	Former smoker	36,342	36.23
	Current smoker	4,713	4.70
	NA	2015	2.01
Education	≤ 12 years	35,558	35.45
	12–16 years	41,180	41.06
	≥ 16 years	21,240	21.18
	NA	2,325	2.32
Marriage status	Married	94,327	94.04
	Unmarried	4,264	4.25
	NA	1712	1.71
Income	< 4 million yen	36,777	36.67
	4–6 million yen	30,234	30.14
	≥ 6 million yen	24,495	24.42
	NA	8,797	8.77
Body mass index	BMI < 25	89,474	89.20
	BMI ≥ 25	10,696	10.66
	NA	133	0.13
Maternal medical history			
ADHD	No	98,990	98.69
	Yes	30	0.03
	NA	1,283	1.28
Learning disorder*	No	99,004	98.70
	Yes	16	0.02
	NA	1,283	1.28
Autism, Asperger syndrome, or pervasive developmental disorder (PDD)	No	99,000	98.70
	Yes	20	0.02
	NA	1,283	1.28
Developmental disorder (other)	No	99,006	98.71
	Yes	14	0.01
	NA	1,283	1.28
Autism spectrum quotient	<7	93,393	93.11
	> = 7	2,586	2.58
	NA	4,324	4.31
Diabetes	No	98,890	98.59
	Yes	130	0.13
	NA	1,283	1.28
Gestational diabetes	No	97,274	96.98
	Yes	2,736	2.73
	NA	293	0.29
Child characteristics			
Mean birth weight (g ± SD)	x ≥ 2,500	90,663	90.39
	x < 2,500	9,275	9.25
	NA	365	0.36
Parity	Singleton	98,412	98.11
	Multiple birth	1891	1.89
Sex	Male	51,396	51.24
	Female	48,889	48.74
	Unknown	3	0.00
	NA	15	0.01
Delivery method	Natural vaginal delivery	56,530	56.36
	Induced delivery, vacuum extraction, forceps delivery, or cesarean delivery	43,217	43.09
	NA	556	0.55
Going to nursery school at 6 months of age	No	86,707	86.45
	Yes	6,508	6.49
	NA	7,088	7.07
Going to nursery school at 1 year of age	No	65,966	65.77
	Yes	24,103	24.03
	NA	10,234	10.20
Going to nursery school at 2 years of age	No	42,941	42.81
	Yes	42,413	42.28
	NA	14,949	14.90
Going to nursery school at 3 years of age	No	29,481	29.39
	Yes	50,355	50.20
	NA	20,467	20.41

SD, Standard deviation; NA, Not answered; *; Learning disorder was based on maternal self-reported medical history; detailed subtypes of learning disorder were not available in the questionnaire.

**Table 2:** Sleep characteristics in the JECS.

Sleep duration category	Children’s age							
	0.5 years	(%)	1 year	(%)	1.5 years	(%)	3 years	(%)
Short duration	15,376	14.78	9,289	8.93	13,186	12.67	6,754	6.49
Recommended duration*	51,384	49.38	54,426	52.30	63,694	61.21	63,179	60.71
Long duration	26,655	25.61	26,764	25.72	11,375	10.93	12,480	11.99
Missing	10,647	10.23	13,583	13.05	15,807	15.19	21,649	20.80

* Recommended durations were as follows: 12–15 h at 0.5 years, 11–14 h at 1 and 1.5 years, and 10–13 h at 3 years (Chaput et al., Nature and Science of Sleep 2018:10421–430).

**Figure 2:**
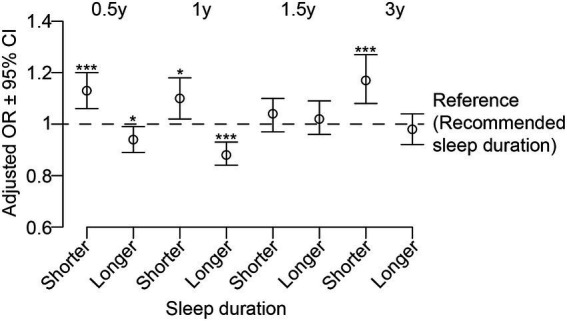
Shorter sleep duration was associated with general child development at 3 years old. Delayed development, as the outcome variable, was defined as a score below the cutoff in more than one category. Sleep duration, as the exposure variable, was defined as “shorter” if it was below the recommended sleep duration and “longer” if it exceeded the recommended sleep duration. The reference category was defined as the recommended sleep duration (0.5 years: 14 h ≤ x < 15 h; 1 year: 13 h ≤ x < 14 h; 1.5 and 3 years: 12 h ≤ x < 13 h). ORs were calculated using multivariate logistic regression analysis. ORs were adjusted for maternal factors (age, marriage status, medical history [ADHD, learning disorder, autism, and other conditions], smoking status, delivery method, education, household income, multiple pregnancy, gestational age, and parity) and child factors (birth weight and sex). *N* at 0.5 years = 63,321; *N* at 1 year = 62,934; *N* at 1.5 years = 62,508; *N* at 3 years = 63,954. Asterisks indicate statistically significant differences compared to the recommended sleep duration group: *: *p* < 0.05; ***: *p* < 0.001.

**Figure 3:**
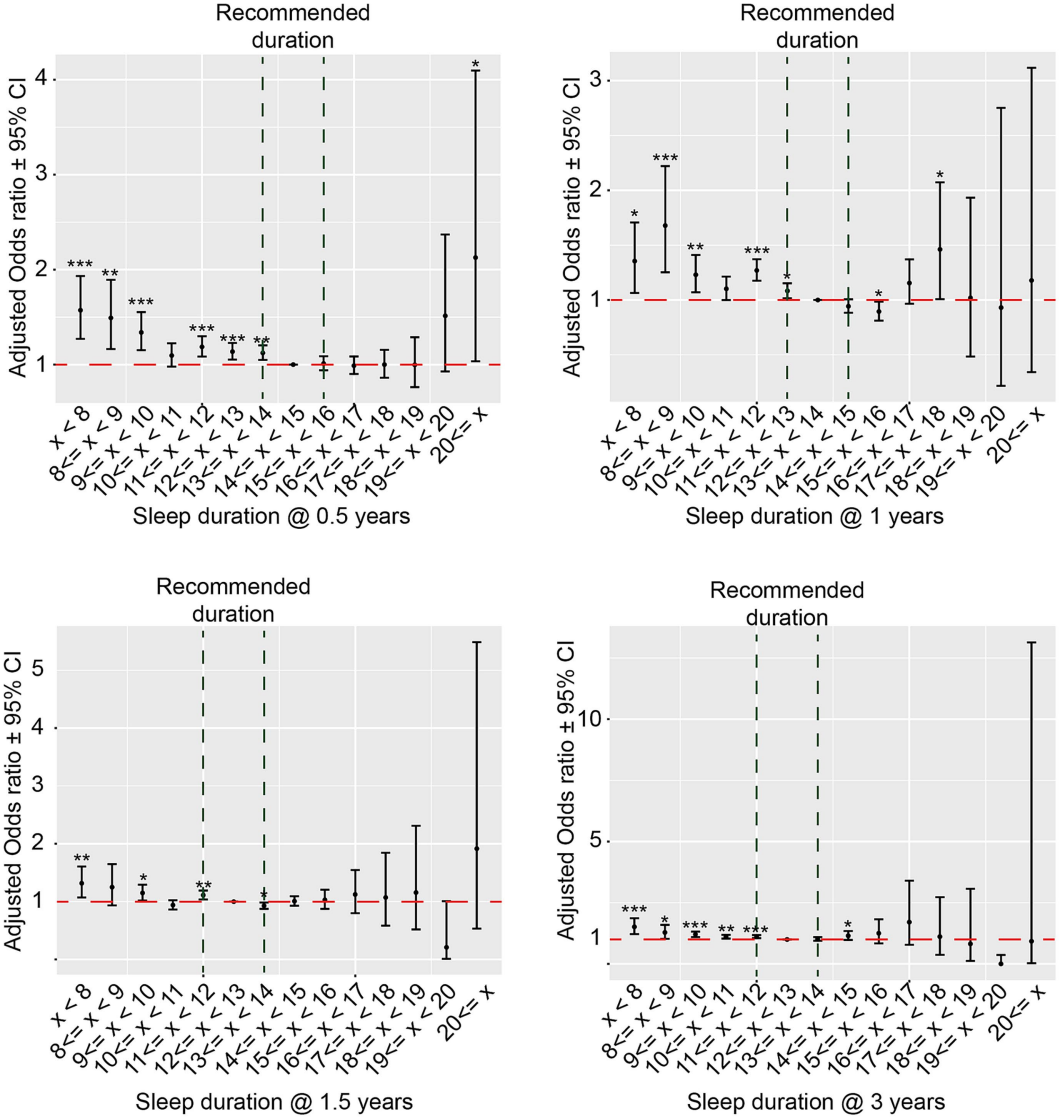
Association between total sleep duration and general child development at 3 years of age. Delayed development, as the outcome variable, was defined as a score below the cutoff in more than one category. Total sleep duration was an exposure variable. The reference category was defined as the recommended sleep duration (0.5 years: 14 h ≤ x < 15 h; 1 year: 13 h ≤ x < 14 h; 1.5 and 3 years: 12 h ≤ x < 13 h). ORs were calculated using multivariate logistic regression analysis. ORs were adjusted for maternal factors (age, marriage status, medical history [ADHD, learning disorder, autism, and other conditions], smoking status, delivery method, education, household income, multiple pregnancy, gestational age, and parity) and child factors (birth weight and sex). *N* at 0.5 years = 63,321; *N* at 1 year = 62,934; *N* at 1.5 years = 62,508; *N* at 3 years = 63,954. Asterisks indicate statistically significant differences compared to the recommended sleep duration group: *: *p* < 0.05; **: *p* < 0.01; ***: *p* < 0.001. The number of participants and exact ORs, 95% CIs, and *p*-values for each sleep duration category are provided in Supplementary Tables 1, 2.

To examine the association between sleep duration and child development, logistic regression analysis was performed with child development at 3 years of age as the dependent variable and sleep duration (shorter, recommended, and longer) and other confounding factors as independent variables. Shorter sleep durations at 0.5, 1, and 3 years of age were associated with child development at 3 years of age (0.5 years: OR = 1.11, *p* < 0.001; 1 year: OR = 1.10, *p* = 0.014; 3 years: OR = 1.15, *p* < 0.001; Figure 2). Longer sleep durations at 0.5 and 1 years of age significantly decreased the odds of developmental delay at 3 years of age (0.5 years: OR = 0.93, *p* = 0.015; 1 year: OR = 0.88, *p* < 0.001; Figure 2). Sleep durations shorter than the recommended range were generally associated with higher odds of developmental delay, particularly at 6 months and 1 year of age (Figure 3; Supplementary Tables 1, 2). The exact ORs, 95% CIs, and *p*-values for each sleep duration category are shown in Supplementary Table 2.

**Figure 4:**
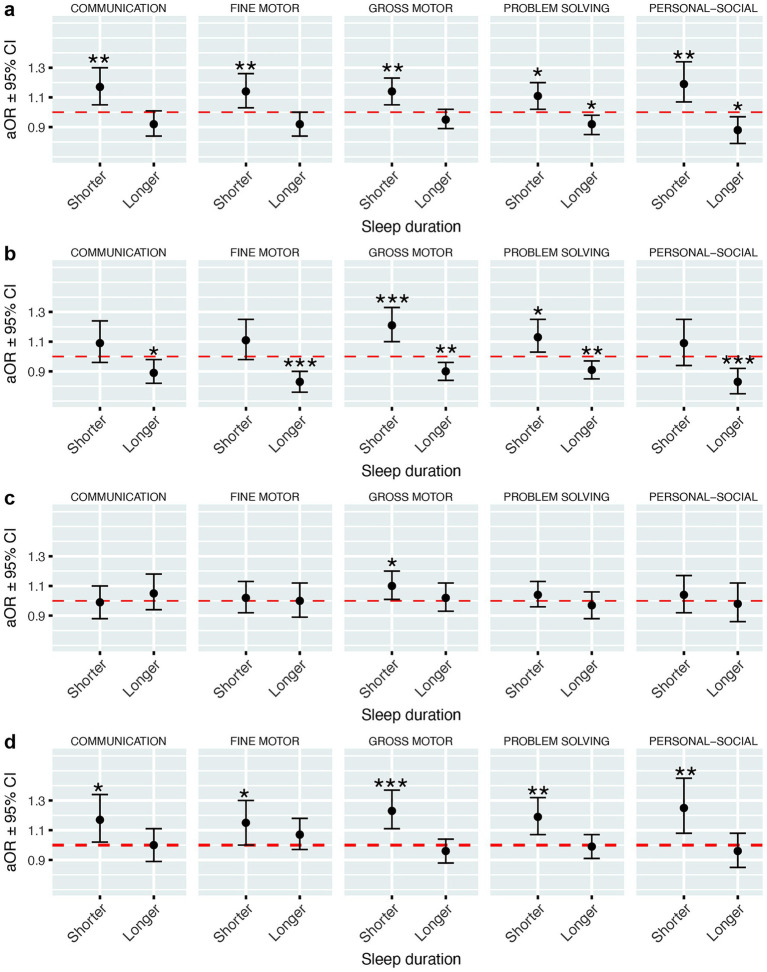
Association between sleep duration at 0.5 years **(a)**, 1 year **(b)**, 1.5 years **(c)**, and 3 years **(d)** and child development at 3 years of age. Delayed development, as the outcome variable, was defined as a score below the cutoff value in each category. Sleep duration, as the exposure variable, was defined as “shorter” if it was below the recommended sleep duration and “longer” if it exceeded the recommended sleep duration. The reference category was defined as the recommended sleep duration. ORs were calculated using multivariate logistic regression analysis. ORs were adjusted for maternal factors (age, marriage status, medical history [ADHD, learning disorder, autism, and other conditions], smoking status, delivery method, education, household income, multiple pregnancy, gestational age, and parity) and child factors (birth weight and sex). *N* at 0.5 years = 63,321; *N* at 1 year = 62,934; *N* at 1.5 years = 62,508; *N* at 3 years = 63,954. Asterisks indicate statistically significant differences compared to the recommended sleep duration group: *: *p* < 0.05; **: *p* < 0.01; ***: *p* < 0.001. The exact ORs, 95% CIs, and *p*-values are shown in Supplementary Table 4.

To elucidate the categories affected by total sleep duration, we analyzed the association between each ASQ-3 category and sleep duration (Figure 4). Shorter sleep durations at 0.5 and 3 years of age were associated with increased odds of developmental delay across all developmental categories. Shorter sleep duration at 1 year of age was significantly associated with increased odds of developmental delay in the gross motor and problem-solving domains. Shorter sleep duration at 1.5 years of age was significantly associated with increased odds of developmental delay in the gross motor domain. In contrast, longer sleep duration was associated with decreased odds of developmental delay in the problem-solving and personal–social domains at 0.5 years of age and in all five developmental domains at 1 year of age. The exact ORs, 95% CIs, and *p*-values for each developmental domain are shown in Supplementary Table 4.

We next examined the association between sleep duration and the number of developmental delay categories using a generalized linear mixed model that included a random intercept for each child (Table 3; Supplementary Table 4). Shorter sleep duration was significantly positively associated with the number of developmental delay categories after accounting for within-child correlations.

**Table 3:** Association between sleep duration and child development adjusted for random effects.

Model	Variables	coef	se (coef)	z	Pr(>|z|)
Crude	Sleep duration	0.046	0.014	3.297	0.001
Model #1	Sleep duration	0.049	0.014	3.376	0.001
	Maternal age	0.047	0.002	29.723	0.000
	Marriage status	0.071	0.041	1.726	0.084
	Smoking status	−0.120	0.014	−8.824	0.000
Model #2	Sleep duration	0.054	0.015	3.709	<0.001
	Maternal age	0.043	0.002	27.320	<0.001
	Marriage status	0.036	0.041	0.876	0.381
	Smoking status	−0.131	0.014	−9.632	<0.001
	AQ10	0.528	0.043	12.358	<0.001
	BMI	0.088	0.025	3.508	<0.001
	Gestational age	0.365	0.037	9.960	<0.001
	Delivery method	0.156	0.015	10.120	<0.001
	Birth weight	0.512	0.028	18.422	<0.001
	Child’s sex	−0.350	0.015	−22.922	<0.001

AQ10, Autism-Spectrum Quotient 10-item version; BMI, body mass index; coef, coefficient; SE, standard error. Pr(>|z|) indicates the two-sided *p*-value for the z statistic.

We also examined the association between sleep environment factors and shorter sleep duration in the Detailed Study sub-cohort. The factors analyzed included pets entering the bedroom, noisy environment, sleep disturbances caused by noise, sleeping place, sleep posture, scheduled bedtime, and asthma. Sleeping place was positively associated with shorter sleep duration (*p* = 0.049), and sleep posture was negatively associated with shorter sleep duration (*p* < 0.001). Pets entering the bedroom, noisy environment, sleep disturbances caused by noise, scheduled bedtime, and asthma were not significantly associated with shorter sleep duration (Table 4). *Post hoc* pairwise comparisons among sleeping place categories did not show statistically significant differences after Tukey’s adjustment.

**Table 4:** Factors influencing shorter sleep duration.

Covariates	coef	se (coef)	z	Pr(>|z|)
Animal entering the bedroom	−0.13	0.17	−0.77	0.44
Noisy environment	0.04	0.12	0.31	0.76
Sleep disturbances caused by noise	−0.07	0.22	−0.31	0.76
Sleeping place	0.13	0.06	1.97	0.049
Sleep posture	−0.10	0.03	−3.54	<0.001
Scheduled bedtime	0.12	0.12	0.97	0.33
Asthma	−0.05	0.15	−0.30	0.77

We conducted three sensitivity analyses. First, in the current study, we did not exclude children with developmental disorders. As some children who experience sleep difficulties have developmental disorders, the results could potentially reflect the influence of developmental disorders on child development rather than the impact of sleep duration. To examine this possibility, we conducted a sensitivity analysis in a sample that excluded children with developmental disorders (Supplementary Figures 1–3, Supplementary Tables 5, 6). The analysis revealed that the results remained the same after removing children with developmental disorders, indicating that shorter sleep duration, rather than developmental disorders, was associated with child development. Second, in a previous study, nighttime awakening was associated with child development. In the current study, we did not include nighttime awakening as a confounding factor in the main analysis. Therefore, we added nighttime awakening as a confounding factor in the logistic regression models (Supplementary Figures 4–6; Supplementary Table 7). Nighttime awakening and shorter sleep duration were both associated with child development (Supplementary Figures 4–6; Supplementary Table 7). Finally, we performed complete case analysis and did not impute missing data. To consider the effects of missing data, we performed multiple imputation before analysis and performed logistic regression and generalized linear mixed model analyses. After handling missing data using multiple imputation, shorter sleep duration showed a tendency to be associated with child development (Supplementary Figures 7–9). However, in the generalized linear mixed model analysis, there was no significant association between shorter sleep duration and child development or between shorter sleep duration and sleeping with parents (Supplementary Tables 8–9). The association was attenuated and no longer statistically significant after multiple imputation, and other covariates in the mixed-effects Poisson models accounted for within-individual correlations.

## 4 Discussion

In this large prospective birth cohort study, shorter sleep duration in early childhood was associated with developmental delay at 3 years of age. This association was observed in both logistic regression analyses and mixed-effects models that accounted for repeated measurements (Table 3; Figures 2–4). These findings suggest that total sleep duration may be relevant to early developmental outcomes, although the causal direction and underlying mechanisms remain uncertain. In contrast, the evidence for an association between the sleep environment and shorter sleep duration was weaker, with sleep location showing only marginal evidence and no significant pairwise differences after adjustment (Table 4).

Our findings are broadly consistent with previous studies linking insufficient sleep to neurodevelopmental outcomes (Table 3, Figures 2–4). In the domain-specific analyses, shorter sleep duration was associated with several developmental domains, particularly at 0.5 and 3 years of age, rather than being limited to a single developmental parameter (Figure 4). Gross motor development showed relatively consistent associations across assessment ages, but associations were also observed for communication, fine motor, problem-solving, and personal–social domains at some ages. These results suggest that shorter sleep duration may be related to broad aspects of early development. Several biological mechanisms may explain this association (37). Interestingly, previous studies have indicated that shorter sleep is associated with alterations in growth hormone secretion, memory consolidation, and downregulated expression of genes related to neurodevelopment, such as those involved in axon guidance and developmental programs (38–41). Taken together with previous findings, our results suggest that insufficient sleep duration in early life may be associated with multiple developmental domains through pathways related to neuronal development.

We also examined factors related to shorter sleep duration using data from the Detailed Study sub-cohort. In this analysis, sleep location showed only marginal evidence of an association with shorter sleep duration, and *post hoc* pairwise comparisons among sleep location categories were not statistically significant after adjustment (Table 4). Therefore, our findings provide limited evidence regarding sleep environment factors related to sleep duration and should not be interpreted as demonstrating a direct association between the sleep environment and child development.

A previous study reported that various factors influence sleep duration, such as environmental noise (42). In contrast, our study did not find a clear association between caregiver-reported environmental noise and shorter sleep duration (Table 4). Several differences in study design may explain this inconsistency. First, our study focused on children younger than 3 years of age, whereas previous studies have often examined older children or hospital/community settings (42, 43). Second, our noise variables were based on caregiver-reported residential environments rather than objective noise measurements. Third, we assessed shorter total sleep duration, whereas other studies may have assessed sleep quality, awakenings, or sleep disturbances. These differences in population, exposure assessment, and outcome definition may have contributed to the inconsistent findings. Further research is required to elucidate the precise effects of environmental noise on child development and sleep duration.

Previous studies have reported both favorable and unfavorable associations of bed-sharing or room-sharing with child sleep and development (12, 19–21). These conflicting results indicate that parent–child bed-sharing and room-sharing remain a controversial topic in parenting research. In the current study, sleeping in a different room showed a trend toward an association with shorter sleep duration, although the evidence was weak. In addition, *post hoc* pairwise comparisons among sleeping place categories were not statistically significant after Tukey’s adjustment, and the association was no longer statistically significant after multiple imputation (Supplementary Table 9). Therefore, the current study provides only limited evidence regarding the effects of parent–child bed-sharing and room-sharing on children’s sleep health. The interpretation of parent–child sleep arrangements requires caution because the same arrangement may have both beneficial and disruptive aspects. Previous studies have reported that approximately 90% of parents and children share the same room in Japan, whereas data collected from Western countries indicate that 35–50% of parents do not sleep with their infants in the same room (44–47). In the current study, more than 90% of participants slept in the same room. This result is consistent with previous reports. Sleeping near parents may support children’s sleep by providing reassurance or facilitating caregiver responses during the night. Conversely, parents tend to put their children to bed first and then complete household tasks or work before going to bed later. In such cases, when parents enter the room or bed, children’s sleep may be interrupted, potentially contributing to nighttime awakening or shorter sleep duration. These mechanisms are not necessarily contradictory, but they indicate that the influence of sleep arrangements may depend on household routines, bedtime timing, and whether the child shares a bed, room, or sleeping space with parents or siblings. Future studies using more granular measures of co-sleeping (e.g., bed-sharing vs. room-sharing and presence of siblings) and lifestyle factors may help clarify how sleep arrangements influence sleep duration and development.

Previous studies have reported an association between nighttime awakening and child development, consistent with the current findings (47–49). However, overall, the literature remains mixed, and recent reviews have noted that associations between infant sleep–wake patterns and cognitive/psychomotor development are not conclusive (18). In the current study, a significant association was observed between shorter sleep duration and child development after adjusting for nighttime awakening. At first glance, nighttime awakening and sleep duration appear to be closely related because increased nighttime awakenings are likely to reduce total sleep duration. It is possible that the impact of shorter sleep duration on child development is partially explained by nighttime awakening. However, because our findings indicated a significant association after adjustment for nighttime awakening, child development may be associated with sleep patterns and sleep duration via other mechanisms or independently. Further studies are needed to clarify the mechanisms underlying these associations.

The results of our sensitivity analyses differed somewhat from those of the primary analyses. Although adjustment for nighttime awakening attenuated the associations, the relationship between shorter sleep duration and development was still present at 0.5 years of age. In addition, because shorter sleep duration was associated with child developmental difficulties, the results suggested that total sleep duration affected child development and that this association persisted after adjustment for nighttime awakening. Multiple imputation analyses showed similar point estimates but wider confidence intervals and fewer significant results, consistent with reduced precision from imputing missing covariates under plausible missing completely at random/missing at random assumptions (Figures 2–4; Table 3; Supplementary Figures 7–9; Supplementary Tables 8, 9). Missing data are unlikely to be missing completely at random, and the observed pattern is more consistent with missing at random. Therefore, although the associations were attenuated and no longer statistically significant in the sensitivity analysis, the direction of the estimates was generally consistent with the primary analysis. Further research is needed to elucidate the details of the association between sleep duration and child development.

The current study has several limitations that should be considered. First, we were not able to measure the quality of sleep, which is known to affect sleep duration. In the current study, we captured only one aspect of sleep, limiting the findings. Second, because we used outcome and confounding factor data provided by caregivers, the data may have been affected by observer bias. For example, a mother may evaluate her child more strictly or generously, which could have negative or positive effects, respectively, on child development data. To address this problem, it would be useful to examine objectively recorded data, such as K-type developmental test results acquired by medically trained staff. Third, we were not able to measure neighbor-generated noise as an environmental factor. Although we assessed perceived environmental noise, such as traffic noise, we did not capture other noise sources, including neighbor-generated or other indoor noise. This is a notable limitation because environmental and neighborhood noise have been reported to disturb sleep, and guidelines on environmental noise emphasize the importance of nighttime noise exposure for sleep health (50). In addition, noise generated by neighbors could potentially influence sleep outcomes (43, 50). Therefore, the lack of information on neighbor-generated noise may have resulted in exposure misclassification and may partly explain the null association between reported environmental noise and shorter sleep duration in this study. To elucidate this issue, further research is needed. Fourth, we captured limited information regarding the sleep environment. For example, sleep environment factors (e.g., whether the infant slept with siblings) and bed-sharing status were not assessed in our study. We only collected information on the sleeping location of children and parents in the questionnaire. However, it is possible that sleeping with siblings affected infants’ sleep. To elucidate this issue, further research is needed. Fifth, we did not collect information on the working status of parents. The number of working women has increased in recent years in Japan. However, childcare leave (generally available for up to 1 year) is institutionalized in Japan, and a previous study reported that 86.6% of employed mothers who gave birth took childcare leave (51). Therefore, parents and children tend to sleep together for less than 1 year. However, it is difficult for women in their 30 s to have shorter sleep duration because of parental behavior (52). Therefore, social and contextual factors, such as working conditions, may influence parent–child sleep arrangements, although these factors were not directly assessed in the current study. Further research is needed to elucidate the precise relationships among parenting, sleep, and parental working status. Sixth, we assessed sleep duration using caregiver reports because of the practical difficulty of monitoring a child’s sleep throughout the night (e.g., checking every 30 min). Thus, concerns remain regarding the reliability of the data collection method. To elucidate this issue, future studies should assess sleep using objective measurement methods such as electroencephalography. Despite these limitations, the current study had two major strengths. First, this study used a large nationwide birth cohort. Thus, we found an association between shorter sleep duration and child development in a sample drawn from the general population in Japan. The second major strength of the current study is the repeated measurement of child development and sleep duration. Using a generalized linear mixed model with repeated measurements enabled us to adjust for individual differences, such as sleep trajectories and developmental characteristics. Thus, the current study was able to reveal an association between shorter sleep duration and child development after adjusting for individual differences. Overall, these findings support an association between shorter sleep duration and child developmental outcomes; however, further studies are needed to clarify causality and underlying mechanisms.

## 5 Conclusion

Sleep duration during early childhood was associated with developmental outcomes at 3 years of age. Sleep location was also related to sleep duration, suggesting that aspects of the sleep environment may influence children’s sleep patterns.

## Data Availability

Data are unsuitable for public deposition due to ethical restrictions and legal framework of Japan. It is prohibited by the Act on the Protection of Personal Information (Act No. 57 of 30 May 2003, amendment on 9 September 2015) to publicly deposit the data containing personal information. Ethical Guidelines for Medical and Health Research Involving Human Subjects enforced by the Japan Ministry of Education, Culture, Sports, Science and Technology and the Ministry of Health, Labour and Welfare also restricts the open sharing of the epidemiologic data. All inquiries about access to data should be sent to: jecs-en@nies.go.jp. The person responsible for handling enquiries sent to this e-mail address is Shoji F. Nakayama, JECS Programme Office, National Institute for Environmental Studies.

## Group member of the Japan Environment and Children’s Study Group

Members of the JECS Group as of 2023: Michihiro Kamijima (principal investigator, Nagoya City University, Nagoya, Japan), Shin Yamazaki (National Institute for Environmental Studies, Tsukuba, Japan), Yukihiro Ohya (National Center for Child Health and Development, Tokyo, Japan), Reiko Kishi (Hokkaido University, Sapporo, Japan), Nobuo Yaegashi (Tohoku University, Sendai, Japan), Koichi Hashimoto (Fukushima Medical University, Fukushima, Japan), Chisato Mori (Chiba University, Chiba, Japan), Shuichi Ito (Yokohama City University, Yokohama, Japan), Zentaro Yamagata (University of Yamanashi, Chuo, Japan), Hidekuni Inadera (University of Toyama, Toyama, Japan), Takeo Nakayama (Kyoto University, Kyoto, Japan), Tomotaka Sobue (Osaka University, Suita, Japan), Masayuki Shima (Hyogo Medical University, Nishinomiya, Japan), Seiji Kageyama (Tottori University, Yonago, Japan), Narufumi Suganuma (Kochi University, Nankoku, Japan), Shoichi Ohga (Kyushu University, Fukuoka, Japan), and Takahiko Katoh (Kumamoto University, Kumamoto, Japan).
