# Supplementary material for: Association between sleep duration and child development: the Japan Environment and Children’s Study
Source: Front Public Health. 2026 Jul 8;14:1736659. doi: 10.3389/fpubh.2026.1736659 (PMC13388294; doi:10.3389/fpubh.2026.1736659)
Supplement: Supplementary file 1 [file Supplementary_file_1.DOCX]

*Supplementary information for*

**Association between sleep duration and child development: the Japan Environment and Children’s Study**

Toshio Masumoto, PhD^1*^, Hiroki Amano, PhD^1^, Shinji Otani, MD, PhD ^2^, Youichi Kurozawa, MD, PhD^1^, Akemi Morita, MD, PhD ^1^ and the Japan Environment and Children’s Study Group

*Corresponding author: Toshio Masumoto

**Supplementary Figure 1**


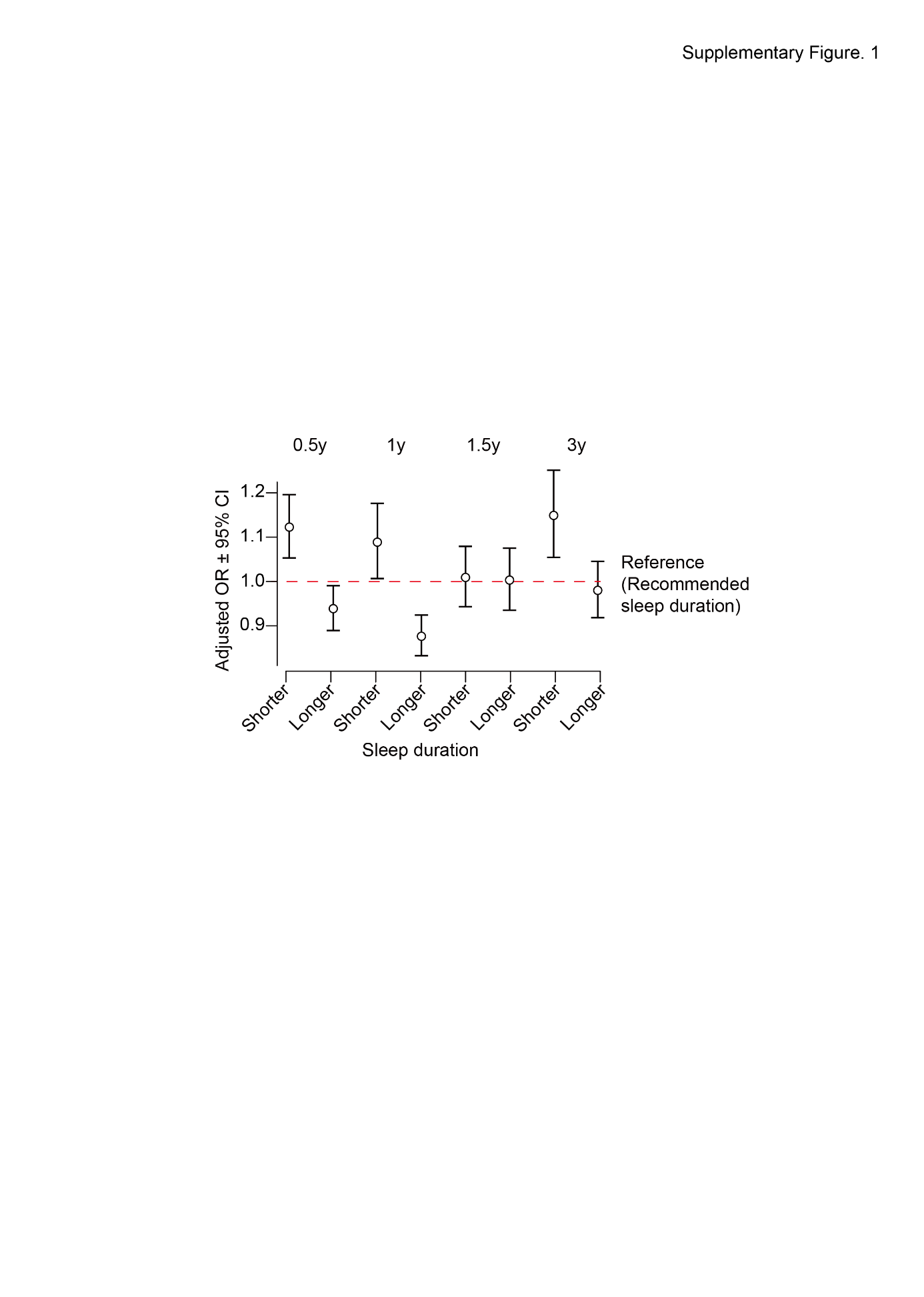


**Supplementary Figure 1. Shorter sleep duration associated with general child development at 3 years old using data which excluded the children with developmental disorder.**

**Supplementary Figure 2.**


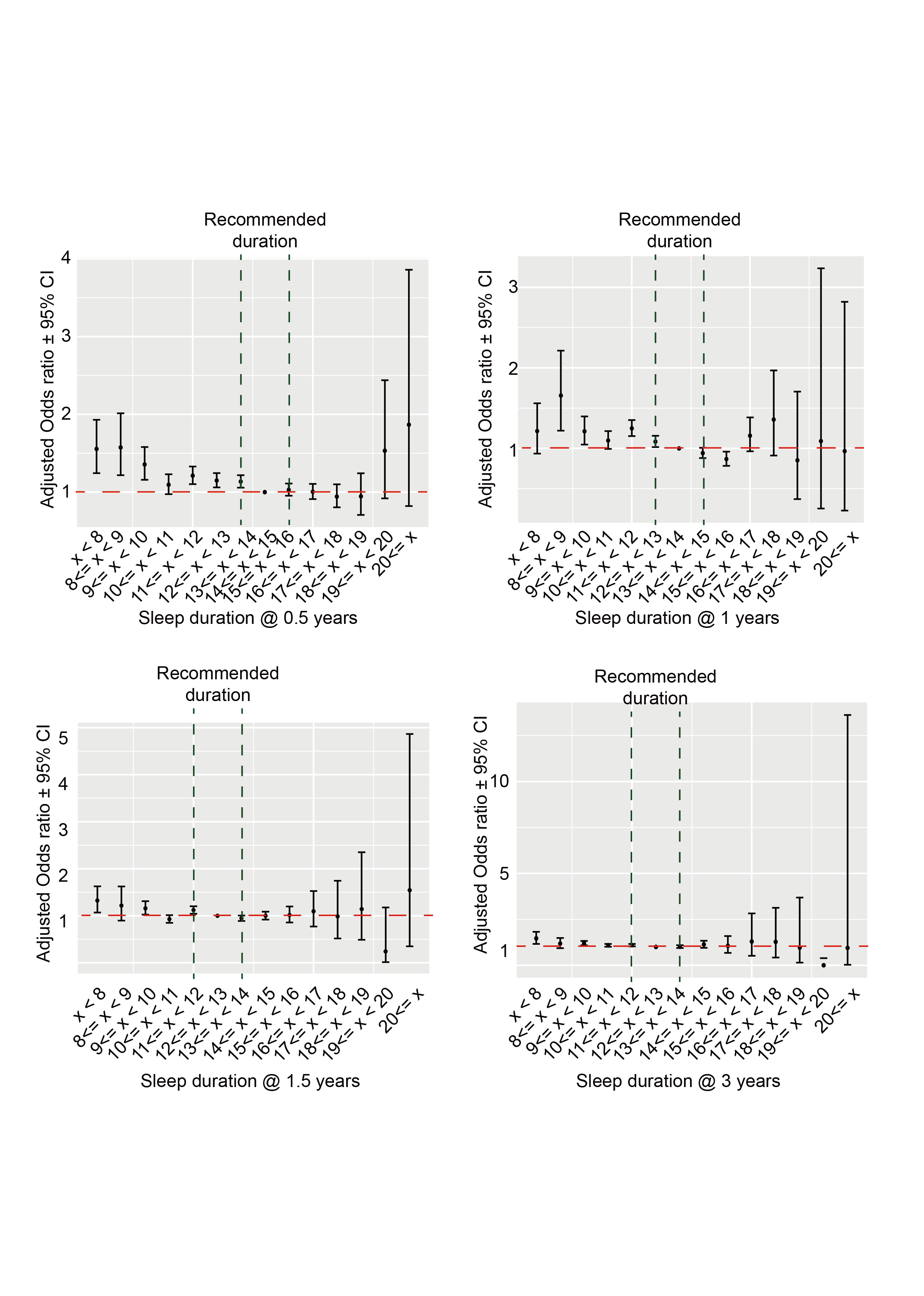


**Supplementary Figure 2. Shorter sleep duration associated with general child development at 3 years old using data which excluded the children with developmental disorder.**

**Supplementary Figure 3**


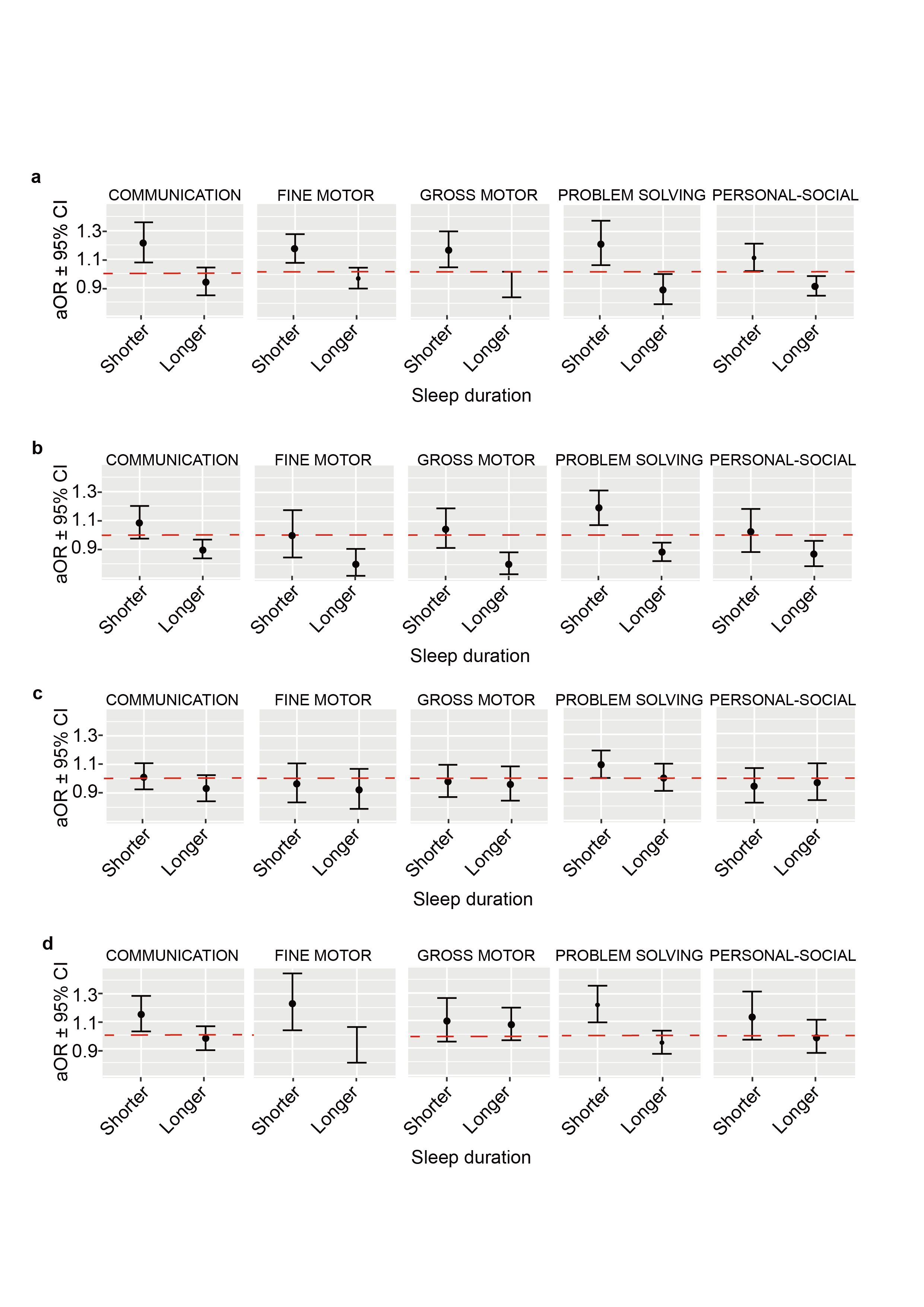


**Supplementary Figure 3. Shorter sleep duration associated with specific child development at 3 years old using data which excluded the children with developmental disorder.**

**Supplementary Figure 4**


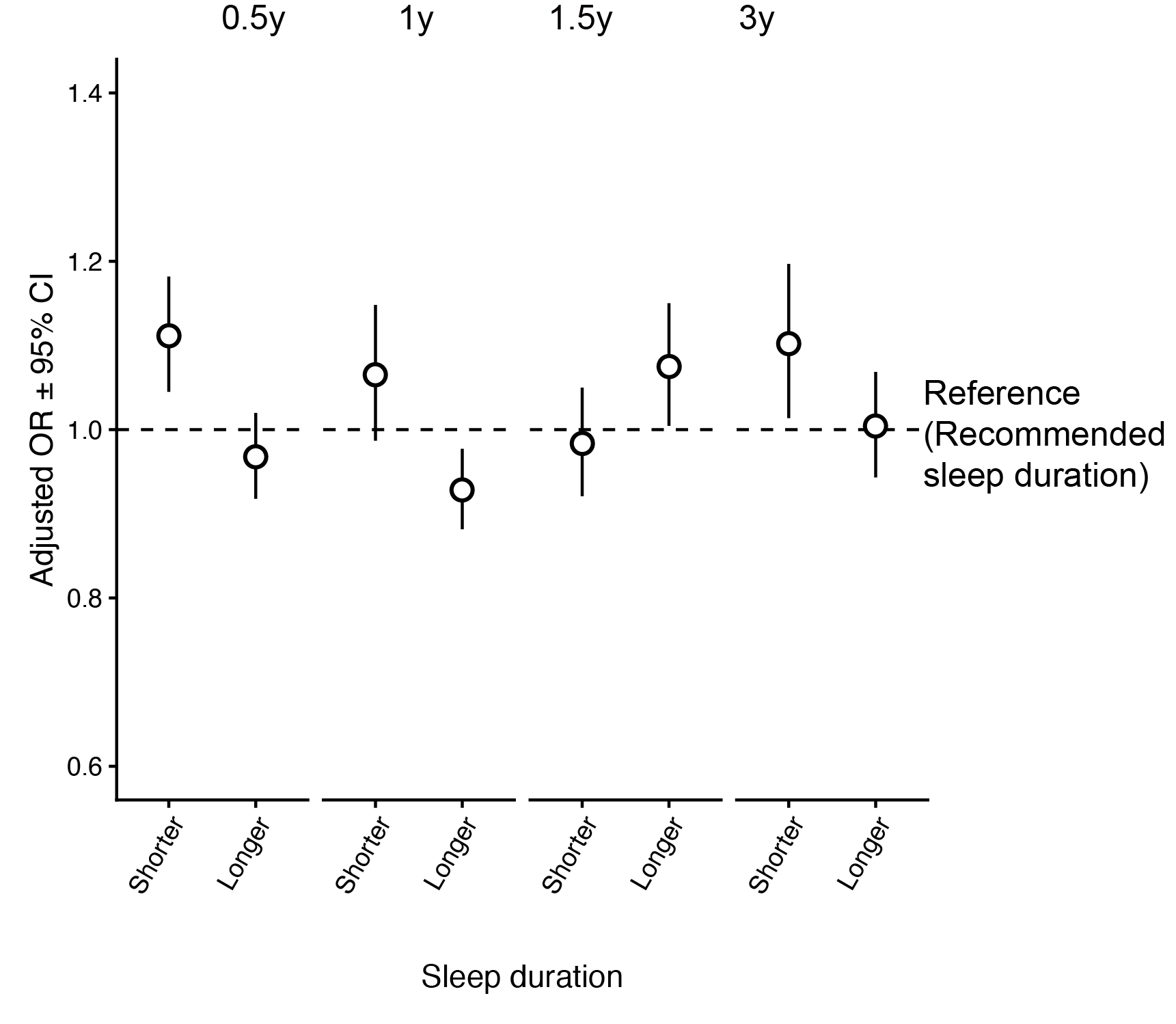


**Supplementary Figure 4. Shorter sleep duration associated with general child development at 3 years old using data including nighttime awaking as confounding factor.**

**Supplementary Figure 5**


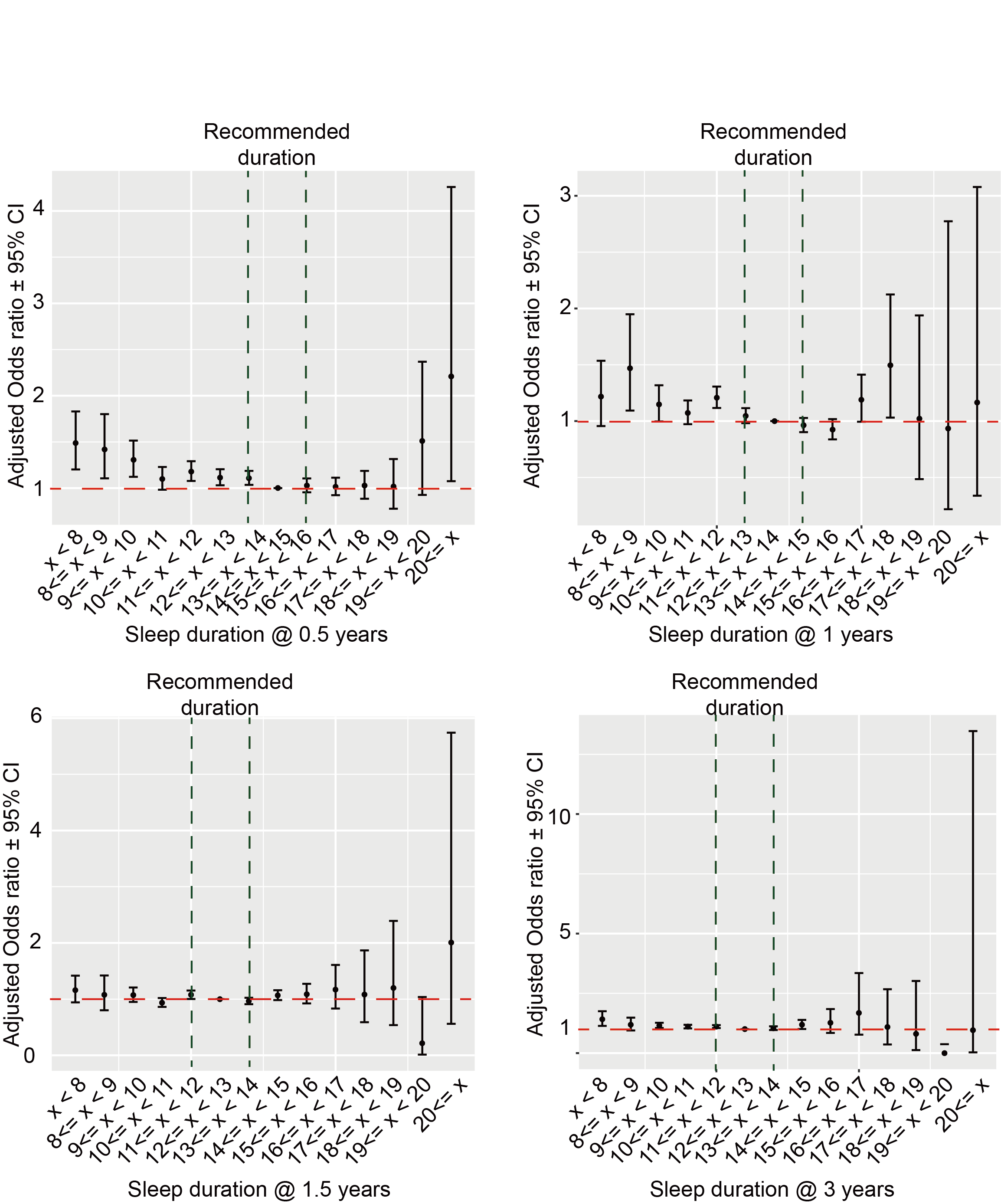
**Supplementary Figure 5. Shorter sleep duration associated with general child development at 3 years old using data including nighttime awaking as confounding factors.**

**.**

**Supplementary Figure 6**


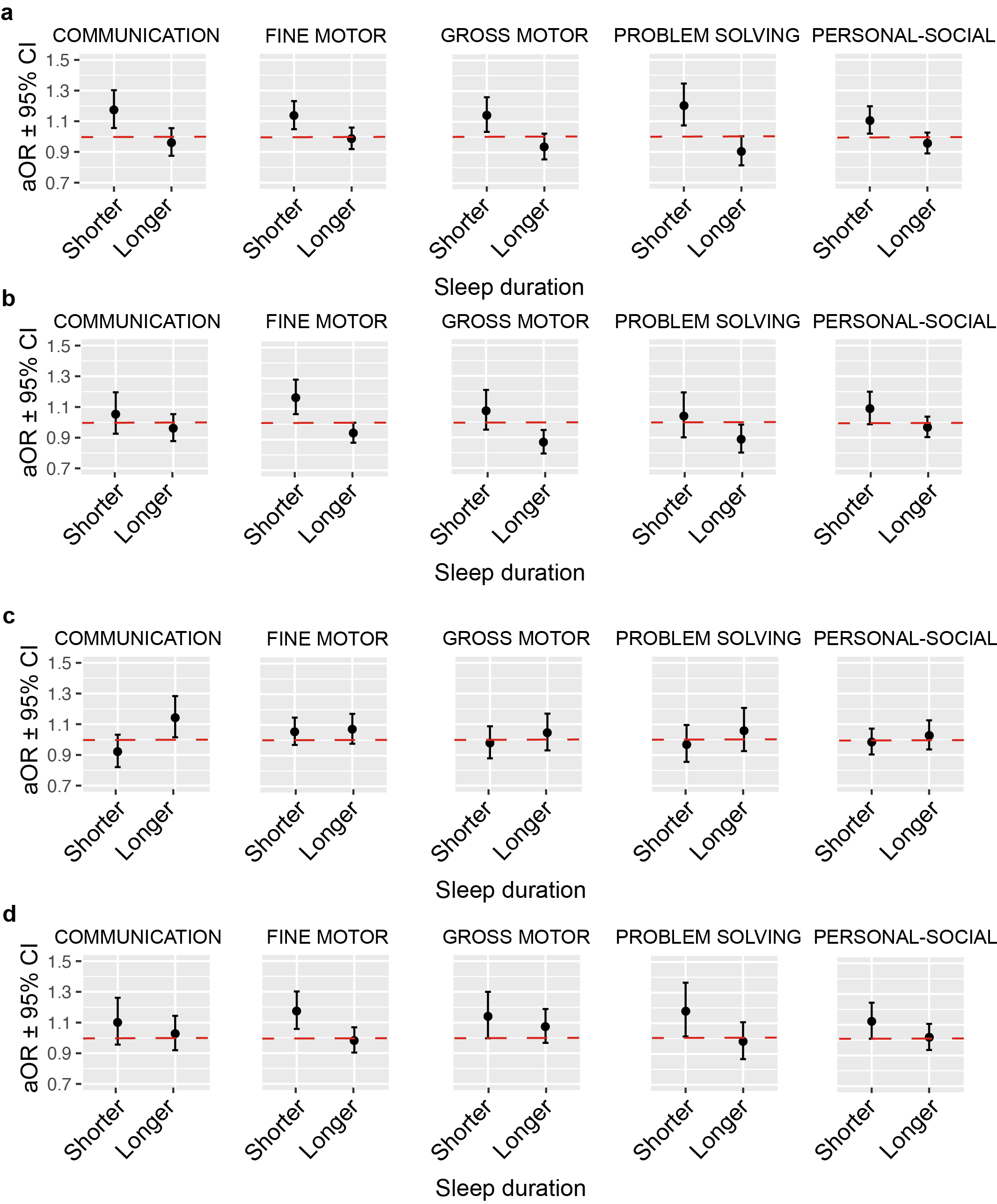


**Supplementary Figure 6. Shorter sleep duration associated with overall child development at 3 years old using data which include nighttime awaking as confounding factor.**

**Supplementary Figure 7**

**
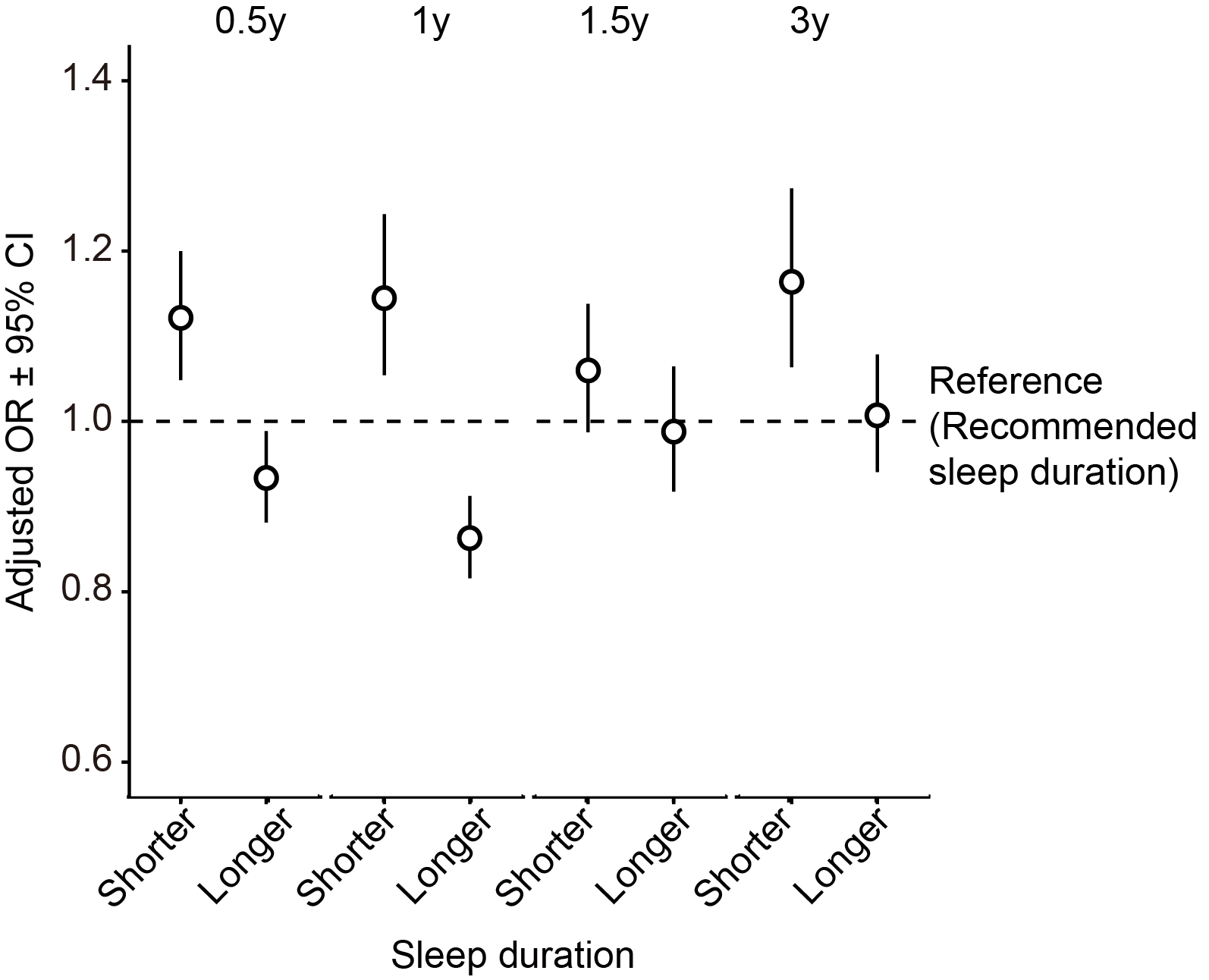
**

**Supplementary Figure 7. Shorter sleep duration associated with general child development at 3 years old using data which imputed missing data.**

**Supplementary Figure 8**


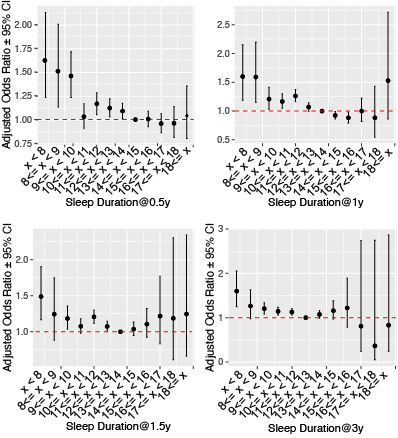


**Supplementary Figure 8. Shorter sleep duration associated with general child development at 3 years old using data which imputed missing data.**

**.**

**Supplementary Figure 9**


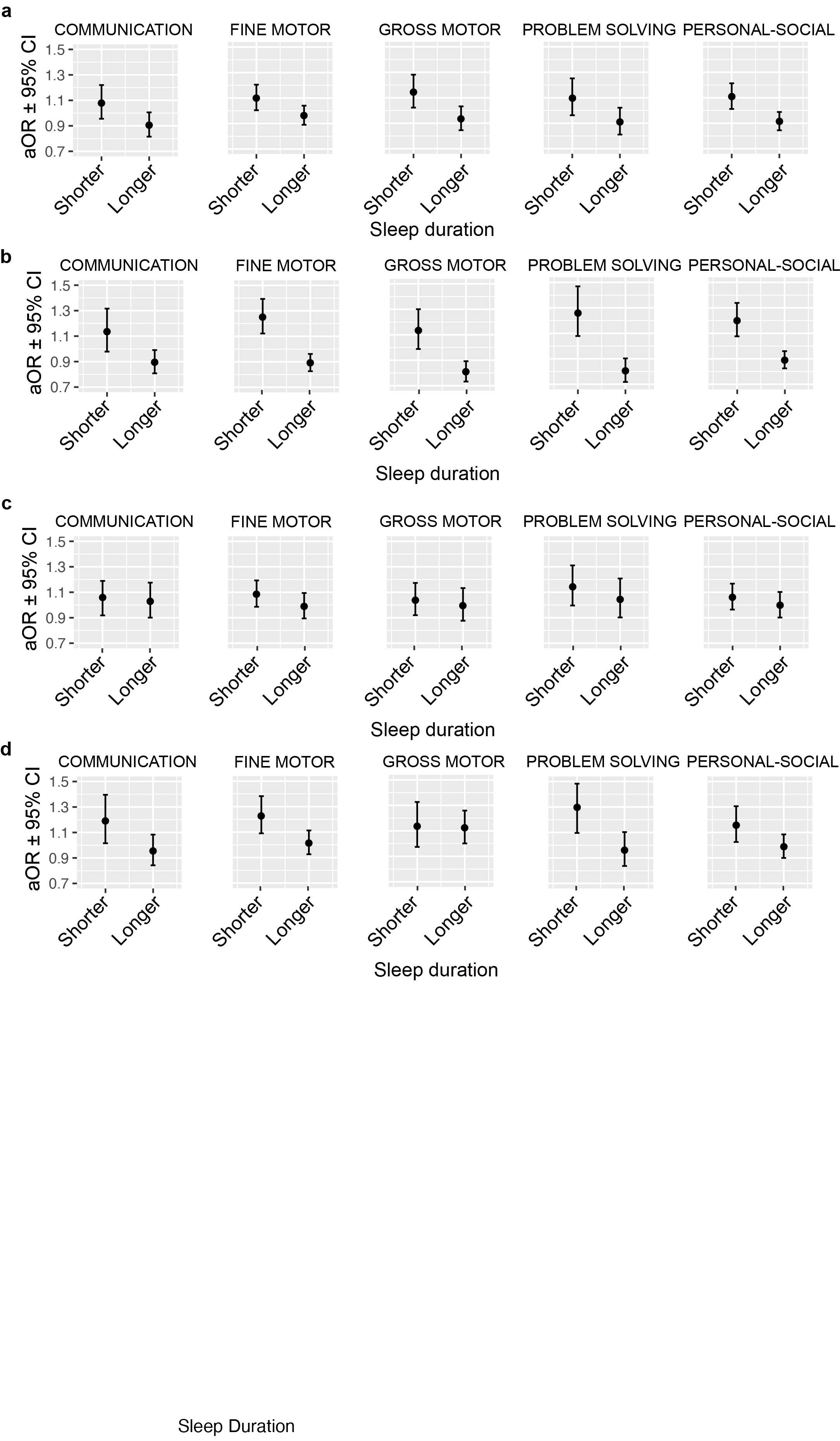


**Supplementary Figure 9. Shorter sleep duration associated with overall child development at 3 years old using data which imputed missing data.**

**.**

| **Supplementary Table1. Number of participants in each sleep-duration category (N)** | | | | | | | | | | | | |  |  |  |
| --- | --- | --- | --- | --- | --- | --- | --- | --- | --- | --- | --- | --- | --- | --- | --- |
| Age | <8h | 8h-9h | 9h-10h | 10h-11h | 11h-12h | 12h-13h | 13h-14h | 14h-15h | 15h-16h | 16h-17h | 17h-18h | 18h-19h | 19h-20h | 20h< | NA* |
| 6m | 1122 | 651 | 2015 | 4370 | 7218 | 11859 | 18548 | 20977 | 15540 | 7668 | 2510 | 678 | 180 | 79 | 6888 |
| 1y | 939 | 427 | 2326 | 5597 | 9202 | 18824 | 26400 | 18455 | 6369 | 1470 | 289 | 99 | 37 | 45 | 9824 |
| 1.5y | 1096 | 460 | 3327 | 8303 | 12480 | 25819 | 25395 | 8937 | 1789 | 384 | 127 | 71 | 36 | 31 | 12048 |
| 3y | 883 | 716 | 5155 | 12528 | 22946 | 27705 | 10547 | 1557 | 249 | 58 | 37 | 18 | 7 | 7 | 17890 |
| *: NA indicate the missing number. | | | | | | | | | |  |  |  |  |  |  |

| **Supplementary Table 2. The exact ORs, 95% CIs, and P-values for each sleep-duration category** | | | |  |
| --- | --- | --- | --- | --- |
| **﻿Assessment age** | **Sleep duration category** | **OR** | **95% CI** | **P-value** |
| 6 months | x < 8 | 1.573 | 1.271, 1.933 | <0.001 |
|  | 8 <= x < 9 | 1.491 | 1.164, 1.893 | 0.001 |
|  | 9 <= x < 10 | 1.340 | 1.152, 1.553 | <0.001 |
|  | 10 <= x < 11 | 1.095 | 0.979, 1.224 | 0.110 |
|  | 11 <= x < 12 | 1.187 | 1.085, 1.299 | <0.001 |
|  | 12 <= x < 13 | 1.137 | 1.053, 1.228 | 0.001 |
|  | 13 <= x < 14 | 1.123 | 1.050, 1.203 | <0.001 |
|  | 14 <= x < 15 (reference) | 1 |  |  |
|  | 15 <= x < 16 | 1.011 | 0.940, 1.088 | 0.767 |
|  | 16 <= x < 17 | 0.990 | 0.901, 1.085 | 0.824 |
|  | 17 <= x < 18 | 1.000 | 0.862, 1.156 | 0.999 |
|  | 18 <= x < 19 | 0.998 | 0.763, 1.288 | 0.989 |
|  | 19 <= x < 20 | 1.513 | 0.928, 2.370 | 0.082 |
|  | 20 <= x | 2.126 | 1.036, 4.097 | 0.030 |
| 1 year | x < 8 | 1.354 | 1.064, 1.706 | 0.012 |
|  | 8 <= x < 9 | 1.678 | 1.252, 2.221 | <0.001 |
|  | 9 <= x < 10 | 1.230 | 1.070, 1.410 | 0.003 |
|  | 10 <= x < 11 | 1.102 | 0.999, 1.214 | 0.050 |
|  | 11 <= x < 12 | 1.270 | 1.176, 1.371 | <0.001 |
|  | 12 <= x < 13 | 1.083 | 1.016, 1.153 | 0.014 |
|  | 13 <= x < 14 (reference) | 1 |  |  |
|  | 14 <= x < 15 | 0.943 | 0.883, 1.006 | 0.077 |
|  | 15 <= x < 16 | 0.895 | 0.812, 0.986 | 0.026 |
|  | 16 <= x < 17 | 1.154 | 0.966, 1.371 | 0.109 |
|  | 17 <= x < 18 | 1.462 | 1.008, 2.073 | 0.039 |
|  | 18 <= x < 19 | 1.019 | 0.484, 1.933 | 0.957 |
|  | 19 <= x < 20 | 0.930 | 0.217, 2.752 | 0.908 |
|  | 20 <= x | 1.179 | 0.342, 3.117 | 0.764 |
| 1.5 years | x < 8 | 1.317 | 1.072, 1.607 | 0.008 |
|  | 8 <= x < 9 | 1.250 | 0.933, 1.649 | 0.124 |
|  | 9 <= x < 10 | 1.149 | 1.019, 1.292 | 0.022 |
|  | 10 <= x < 11 | 0.941 | 0.864, 1.023 | 0.157 |
|  | 11 <= x < 12 | 1.113 | 1.038, 1.192 | 0.003 |
|  | 12 <= x < 13 (reference) | 1 |  |  |
|  | 13 <= x < 14 | 0.928 | 0.875, 0.984 | 0.012 |
|  | 14 <= x < 15 | 1.008 | 0.929, 1.092 | 0.854 |
|  | 15 <= x < 16 | 1.029 | 0.874, 1.205 | 0.728 |
|  | 16 <= x < 17 | 1.123 | 0.799, 1.545 | 0.488 |
|  | 17 <= x < 18 | 1.071 | 0.584, 1.844 | 0.813 |
|  | 18 <= x < 19 | 1.156 | 0.520, 2.311 | 0.699 |
|  | 19 <= x < 20 | 0.209 | 0.012, 1.008 | 0.127 |
|  | 20 <= x | 1.915 | 0.533, 5.485 | 0.262 |
| 3 years | x < 8 | 1.514 | 1.218, 1.868 | <0.001 |
|  | 8 <= x < 9 | 1.284 | 1.024, 1.595 | 0.027 |
|  | 9 <= x < 10 | 1.204 | 1.096, 1.321 | <0.001 |
|  | 10 <= x < 11 | 1.110 | 1.037, 1.188 | 0.003 |
|  | 11 <= x < 12 | 1.115 | 1.054, 1.179 | <0.001 |
|  | 12 <= x < 13 (reference) | 1 |  |  |
|  | 13 <= x < 14 | 1.020 | 0.948, 1.097 | 0.589 |
|  | 14 <= x < 15 | 1.148 | 0.977, 1.343 | 0.089 |
|  | 15 <= x < 16 | 1.254 | 0.838, 1.824 | 0.252 |
|  | 16 <= x < 17 | 1.705 | 0.784, 3.400 | 0.150 |
|  | 17 <= x < 18 | 1.116 | 0.372, 2.728 | 0.826 |
|  | 18 <= x < 19 | 0.817 | 0.126, 3.070 | 0.794 |
|  | 19 <= x < 20 | 0.000 | 0.000, 0.367 | 0.866 |
|  | 20 <= x | 0.928 | 0.028, 13.140 | 0.959 |

| Supplementary Table 3. The exact ORs, 95% CIs, and P-values for each developmental domain | | | |  |  |
| --- | --- | --- | --- | --- | --- |
| Sleep assessment age | ASQ-3 domain | Sleep duration category | OR | 95% CI | P-value |
| 6 months | Communication | Shorter | 1.17 | 1.05–1.30 | 0.003 |
|  |  | Longer | 0.92 | 0.84–1.01 | 0.091 |
|  | Fine motor | Shorter | 1.14 | 1.03–1.26 | 0.009 |
|  |  | Longer | 0.92 | 0.84–1.00 | 0.053 |
|  | Gross motor | Shorter | 1.14 | 1.05–1.23 | 0.001 |
|  |  | Longer | 0.95 | 0.89–1.02 | 0.193 |
|  | Problem solving | Shorter | 1.11 | 1.02–1.20 | 0.012 |
|  |  | Longer | 0.92 | 0.85–0.98 | 0.013 |
|  | Personal-social | Shorter | 1.19 | 1.07–1.34 | 0.002 |
|  |  | Longer | 0.88 | 0.79–0.97 | 0.014 |
| 1 year | Communication | Shorter | 1.09 | 0.96–1.24 | 0.169 |
|  |  | Longer | 0.89 | 0.82–0.98 | 0.015 |
|  | Fine motor | Shorter | 1.11 | 0.98–1.25 | 0.092 |
|  |  | Longer | 0.83 | 0.76–0.90 | <0.001 |
|  | Gross motor | Shorter | 1.21 | 0.99–1.33 | 0.053 |
|  |  | Longer | 0.90 | 0.84–0.96 | 0.003 |
|  | Problem solving | Shorter | 1.13 | 1.03–1.25 | 0.012 |
|  |  | Longer | 0.91 | 0.85–0.97 | 0.006 |
|  | Personal-social | Shorter | 1.09 | 0.94–1.25 | 0.244 |
|  |  | Longer | 0.83 | 0.75–0.92 | <0.001 |
| 1.5 years | Communication | Shorter | 0.99 | 0.88–1.10 | 0.816 |
|  |  | Longer | 1.05 | 0.94–1.18 | 0.389 |
|  | Fine motor | Shorter | 1.02 | 0.92–1.13 | 0.723 |
|  |  | Longer | 1.00 | 0.89–1.12 | 0.998 |
|  | Gross motor | Shorter | 1.10 | 1.01–1.20 | 0.022 |
|  |  | Longer | 1.02 | 0.93–1.12 | 0.640 |
|  | Problem solving | Shorter | 1.04 | 0.96–1.13 | 0.345 |
|  |  | Longer | 0.97 | 0.88–1.06 | 0.504 |
|  | Personal-social | Shorter | 1.04 | 0.92–1.17 | 0.569 |
|  |  | Longer | 0.98 | 0.86–1.12 | 0.795 |
| 3 years | Communication | Shorter | 1.17 | 1.02–1.34 | 0.021 |
|  |  | Longer | 1.00 | 0.89–1.11 | 0.960 |
|  | Fine motor | Shorter | 1.15 | 1.00–1.30 | 0.041 |
|  |  | Longer | 1.07 | 0.97–1.18 | 0.190 |
|  | Gross motor | Shorter | 1.23 | 1.11–1.37 | <0.001 |
|  |  | Longer | 0.96 | 0.88–1.04 | 0.314 |
|  | Problem solving | Shorter | 1.19 | 1.07–1.32 | 0.001 |
|  |  | Longer | 0.99 | 0.91–1.07 | 0.797 |
|  | Personal-social | Shorter | 1.25 | 1.08–1.45 | 0.002 |
|  |  | Longer | 0.96 | 0.85–1.08 | 0.470 |
| *The reference category was the recommended sleep duration group at each assessment age.* | | | |  |  |

**Supplementary Table 4 Pearson’s Correlation Matrix in Model1 and 2**

| Model1 | age | marriage | smoking | ASQ3 | Sleep duration |
| --- | --- | --- | --- | --- | --- |
| age | 1 | -0.1663485 | -0.0490467 | 0.04127288 | -0.035116 |
| marriage | -0.1663485 | 1 | 0.0962642 | 0.01133451 | 0.00532693 |
| smoking | -0.0490467 | 0.0962642 | 1 | 0.00042661 | 0.00542945 |
| ASQ3 | 0.04127288 | 0.01133451 | 0.00042661 | 1 | -0.01932 |
| Sleep duration | -0.035116 | 0.00532693 | 0.00542945 | -0.01932 | 1 |

| Model2 | age | marriage | smoking | ASQ3 | Sleep duration | Gestational age | Mother's BMI | Method of delivery | AQ10 | Birth Weight | Child's Sex |
| --- | --- | --- | --- | --- | --- | --- | --- | --- | --- | --- | --- |
| age | 1 | -1.62E-01 | -0.0493419 | 0.04153389 | -0.0358617 | 0.03346026 | 4.44E-02 | 0.21653154 | -2.14E-02 | 0.02673585 | -0.0010326 |
| marriage | -0.1621295 | 1.00E+00 | 0.09503816 | 0.0114424 | 0.00742556 | -0.0022341 | 5.84E-05 | -0.1513312 | 1.43E-02 | 0.00519987 | -0.0091471 |
| smoking | -0.0493419 | 9.50E-02 | 1 | 0.00067823 | 0.00606557 | 0.01174549 | 5.20E-02 | 0.04675499 | 1.08E-02 | 0.01459226 | -0.0011607 |
| ASQ3 | 0.04153389 | 1.14E-02 | 0.00067823 | 1 | -0.0195425 | 0.07282962 | 3.84E-02 | -0.0246452 | 4.56E-02 | 0.06434991 | -0.1379511 |
| Sleep duration | -0.0358617 | 7.43E-03 | 0.00606557 | -0.0195425 | 1 | 0.00871074 | 1.28E-02 | -0.0160334 | -8.08E-03 | 0.00450279 | -0.0038912 |
| Gestational age | 0.03346026 | -2.23E-03 | 0.01174549 | 0.07282962 | 0.00871074 | 1 | 2.61E-02 | -0.013127 | 1.88E-03 | 0.45405995 | -0.0238146 |
| Mother's BMI | 0.04437027 | 5.84E-05 | 0.05203988 | 0.03844554 | 0.01279308 | 0.02612184 | 1.00E+00 | 0.03660392 | -3.80E-05 | -0.0071241 | -0.0057653 |
| Method of delivery | 0.21653154 | -1.51E-01 | 0.04675499 | -0.0246452 | -0.0160334 | -0.013127 | 3.66E-02 | 1 | -1.93E-02 | -0.0480855 | -0.0030714 |
| AQ10 | -0.0214067 | 1.43E-02 | 0.01084816 | 0.0456325 | -0.0080764 | 0.00188255 | -3.80E-05 | -0.0193428 | 1.00E+00 | 0.00429902 | 0.00014326 |
| Birth Weight | 0.02673585 | 5.20E-03 | 0.01459226 | 0.06434991 | 0.00450279 | 0.45405995 | -7.12E-03 | -0.0480855 | 4.30E-03 | 1 | 0.04190042 |
| Child's Sex | -0.0010326 | -9.15E-03 | -0.0011607 | -0.1379511 | -0.0038912 | -0.0238146 | -5.77E-03 | -0.0030714 | 1.43E-04 | 0.04190042 | 1 |

| Supplementary Table 5. Association between sleep duration and child development adjusted for random effects removing autism traits | | | | | |
| --- | --- | --- | --- | --- | --- |
|  |  | coef | se(coef) | z | Pr(>\|z\|) |
| *Crude* |  |  |  |  |  |
|  | Sleep Duration | 0.033 | 0.014 | 2.331 | 0.020 |
| *Model#1* |  |  |  |  |  |
|  | Sleep Duration | 0.026 | 0.015 | 1.796 | 0.073 |
|  | Mother's Age | 0.043 | 0.002 | 27.314 | 0.000 |
|  | Marriage Status | 0.083 | 0.041 | 2.019 | 0.044 |
|  | Smoking Status | -0.119 | 0.014 | -8.819 | 0.000 |
| *Model #2* |  |  |  |  |  |
|  | Sleep Duration | 0.028 | 0.015 | 1.918 | 0.055 |
|  | Mother's Age | 0.280 | 0.036 | 7.672 | 0.000 |
|  | Marriage Status | 0.108 | 0.025 | 4.379 | 0.000 |
|  | Smoking Status | 0.143 | 0.015 | 9.489 | 0.000 |
|  | AQ10 | 0.537 | 0.042 | 12.679 | 0.000 |
|  | BMI | 0.040 | 0.002 | 25.323 | 0.000 |
|  | Gestational age | 0.040 | 0.041 | 0.967 | 0.334 |
|  | Method of delivery | -0.131 | 0.014 | -9.675 | 0.000 |
|  | Birth Weight | 0.480 | 0.028 | 17.412 | 0.000 |
|  | Child's Sex | -0.367 | 0.015 | -24.606 | 0.000 |

| Supplementary Table 6. Factors in shorter sleep duration | | | |  |
| --- | --- | --- | --- | --- |
|  |  |  |  |  |
| Covariates | coef | se(coef) | z | Pr(>\|z\|) |
| Animal entering the bedroom | -0.12 | 0.17 | -0.67 | 0.50 |
| Noisy environment | 0.05 | 0.12 | 0.40 | 0.69 |
| Sleep disturbance caused by noise | -0.02 | 0.23 | -0.08 | 0.94 |
| Sleep place | 0.11 | 0.07 | 1.76 | 0.08 |
| Sleep posture | -0.10 | 0.03 | -3.68 | 0.00 |
| Scheduled sleep start | 0.10 | 0.12 | 0.80 | 0.42 |
| Asthma | -0.05 | 0.16 | -0.31 | 0.76 |

| Supplementary Table 7. Association between sleep duration and child development adjusted for random effects | | | | | |
| --- | --- | --- | --- | --- | --- |
|  |  | coef | se(coef) | z | Pr(>\|z\|) |
| *Crude* |  |  |  |  |  |
|  | Sleep Duration | 0.046 | 0.014 | 3.297 | 0.001 |
| *Model#1* |  |  |  |  |  |
|  | Sleep Duration | 0.018 | 0.015 | 1.238 | 0.216 |
|  | Night_time_waking_number | 0.086 | 0.006 | 14.164 | 0.000 |
|  | Mother's Age | 0.047 | 0.002 | 29.708 | 0.000 |
|  | Marriage Status | 0.072 | 0.041 | 1.760 | 0.079 |
|  | Smoking Status | -0.120 | 0.014 | -8.849 | 0.000 |
| *Model #2* |  |  |  |  |  |
|  | Sleep Duration | 0.024 | 0.015 | 1.603 | 0.109 |
|  | Night_time_waking_number | 0.086 | 0.006 | 13.944 | 0.000 |
|  | Mother's Age | 0.043 | 0.002 | 27.296 | 0.000 |
|  | Marriage Status | 0.037 | 0.041 | 0.900 | 0.368 |
|  | Smoking Status | -0.132 | 0.014 | -9.661 | 0.000 |
|  | AQ10 | 0.528 | 0.043 | 12.352 | 0.000 |
|  | BMI | 0.089 | 0.025 | 3.534 | 0.000 |
|  | Gestational age | 0.367 | 0.037 | 10.000 | 0.000 |
|  | Method of delivery | 0.156 | 0.015 | 10.092 | 0.000 |
|  | Birth Weight | 0.513 | 0.028 | 18.453 | 0.000 |
|  | Child's Sex | -0.350 | 0.015 | -22.916 | 0.000 |

Supplementary Table 8. Association between sleep duration and child development adjusted for random effects using imputed dataset

|  | **Crude IRR (95%CI)** | **Adjusted 1 IRR (95%CI)** | **Adjusted 2 IRR (95%CI)** |
| --- | --- | --- | --- |
| Shorter Sleep duration | 1.008 (0.981–1.036) | 1.007 (0.98–1.036) | 1.007 (0.98–1.035) |
| *: Model 1 adjusted for mother's status (age, marriage status, and smoking status). | | | |
| **: Model 2 adjusted for mother's factors (age, marriage status, smoking, AQ10 score, BMI), gynecologic factors (method of delivery and gestational age) and child's factors (sex, birth weight). | | | |

| Supplementary Table 9. Factors in shorter sleep duration | | | |  |
| --- | --- | --- | --- | --- |
| Covariates | coef | se(coef) | CI_low | CI_high |
| Animal entering the bedroom | -0.083 | 0.14 | -0.36 | 0.20 |
| Noisy environment | 0.024 | 0.10 | -0.17 | 0.21 |
| Sleep disturbance caused by noise | -0.057 | 0.19 | -0.42 | 0.32 |
| Sleep place | 0.095 | 0.06 | -0.01 | 0.20 |
| Sleep posture | **-0.070** | 0.02 | -0.12 | -0.02 |
| Scheduled sleep start | 0.106 | 0.10 | -0.08 | 0.32 |
| Asthma | -0.019 | 0.14 | -0.29 | 0.25 |
